# Poster Session II - A203 EXPERIENCE FROM A SINGLE-CENTRE MULTIDISCIPLINARY EOE CLINIC

**DOI:** 10.1093/jcag/gwaf042.203

**Published:** 2026-02-13

**Authors:** N Ahmed, A Alenezi, A Pedicelli, M Eldaba, N Tardio, W Afif

**Affiliations:** McGill University, Montreal, QC, Canada; McGill University, Montreal, QC, Canada; McGill University, Montreal, QC, Canada; McGill University, Montreal, QC, Canada; McGill University, Montreal, QC, Canada; McGill University, Montreal, QC, Canada

## Abstract

**Aims:**

EoE is an increasingly prevalent condition causing significant morbidity if left untreated. A multidisciplinary clinic including allergists and gastroenterologists has been established at our centre for the management of this condition.

**Methods:**

63 consecutive patients seen in the multidisciplinary EoE clinic between October 2024 and June 2025 were included in this analysis. Patients were referred by their general GI, allergy, or family physician who was following them for EoE.

**Results:**

Median patient age was 30 (range 18-66). Most (46/63, 73%) patients were male. Allergic rhinitis was the most common comorbidity present in 37/63, 58.7%, followed by food allergy (26/63, 41.3%), asthma (24/63, 38.1%) and eczema (14/63, 22.2%). The most common symptoms at presentation were esophageal dysphagia (53/63, 84.1%) and food bolus impaction (38/63, 60.3%). Less common symptoms were heartburn (17/63, 27.0%), chest pain (10/63, 15.9%), regurgitation (10/63, 15.9%), and abdominal pain (6/63, 9.5%). 2 patients (3.2%) had a history of esophageal perforation.

Median I-SEE score at diagnosis was 6 (range 2-40). Median time to diagnosis from onset of symptoms was 41 months (Range 0-24 years). Median EREFS score on initial endoscopy was 2 (range 0-7), median eosinophil count was 40 per high-power field (hpf) (range 0–100). Initial treatments included PPI monotherapy (42/63, 66.7%), PPI with topical steroid (16/63, 25.4%), exclusion diet (8/63, 7.9%), topical steroid alone (3/63, 4.8%), and monitoring (1/63, 1.6%). 4/63 (6.3%) had previously undergone esophageal dilatation.

At the most recent visit, the median follow-up time was 36 months (range 0–23 years) from diagnosis. Treatment had been changed in 37/63 (58.7%) patients. At follow up, 26/63 (41.3%) were on PPI and topical steroid, 21/63 (33.3%) on PPI alone, 11/63 (17.5%) on topical steroid alone, 5/63 (7.9%) on dupilumab, and 3/63 (4.8%) on monitoring. At follow up, median I-SEE score was 3 (Range 0-19). Median EREFS score on most recent endoscopy was 2 (Range 0-6), median eosinophil count was 19/hpf (Range 0-200). There was a significant reduction in I-SEE, EREFS and eosinophil count at follow up (p < 0.001, =0.033, =0.022) respectively.

**Conclusions:**

Patients from our dedicated EoE clinic showed significant improvement in symptoms and standardized outcomes during the follow up period. Prompt optimization of EoE treatment is necessary to prevent the development of irreversible complications, and most patients in this cohort required second-line treatment, including escalation to biologic therapy. Dedicated multidisciplinary clinics have been shown to improve clinical outcomes in patients with other chronic conditions such as IBD and CLD. Similarly, dedicated EoE clinics may have the potential to improve clinical outcomes by facilitating optimization of treatment and multidisciplinary follow-up.

**Funding Agencies:**

None

